# Oral Mycobiome: Composition, Functionality and Clinical Implication

**DOI:** 10.3390/jof12070528

**Published:** 2026-07-17

**Authors:** Geovani Moreira da Cruz, Amanda Siqueira Fraga, Maíra Terra Garcia, Juliana Campos Junqueira

**Affiliations:** Department of Biosciences and Oral Diagnosis, Institute of Science and Technology, São Paulo State University (UNESP), São José dos Campos 12245-000, SP, Brazil; geovani.moreira@unesp.br (G.M.d.C.); maira.garcia@unesp.br (M.T.G.)

**Keywords:** dysbiosis, human microbiome, mycobiome, microbiota, oral cavity, oral fungal microbiome

## Abstract

Historically, the study of oral fungal species was limited by the inability to cultivate most of them. However, advances in metagenomic techniques have enabled the direct identification of microbial genomes from human samples, markedly broadening our understanding of the oral mycobiome. This narrative review aims to analyze the available scientific evidence on the composition and dynamics of the oral mycobiome, as well as its influence on the development of local pathological conditions. The oral mycobiome is highly diverse, with emphasis on genus *Candida*, followed by *Cladosporium*, *Aureobasidium* and *Saccharomyces. Candida albicans* remains the most frequently identified species in both health and diseases state. However, individuals with oral candidiasis present a higher detection of *Candida dubliniensis*, *Candida parapsilosis*, *Pichia kudriavzevii*, *Antrodiella micra* and *Cladosporium sphaerospermum*. In dental caries, *C. albicans* and *C. dubliniensis* are associated with advanced lesions, whereas *Debaryomyces* and *Rhodotorula* may exert protective effects against *Streptococcus mutans*, a cariogenic bacterium. In periodontitis, an increase in yeast-bacteria interactions is observed. Additionally, *C. albicans* has been implicated in oral carcinogenesis through multiple mechanisms. These findings highlight the need for a deeper understanding of the oral mycobiome to enable early detection of oral diseases and the development of therapeutic approaches.

## 1. Introduction

The oral cavity hosts a high quantity of microorganisms, second only to the gastrointestinal tract. This oral microbial community is diverse and includes bacteria, fungi, viruses and archaea, which coexist in dynamic equilibrium and play important roles in maintaining oral and systemic health [1,2]. However, microbe–host interactions can be constantly affected by lifestyle habits and environmental factors, causing changes in the microbial groups that normally predominate, leading to an imbalance that favors the emergence of diseases, a condition known as dysbiosis [3].

Many of these microorganisms cannot be easily cultivated in the laboratory, which has historically limited their study. With the advancement of metagenomic techniques, it has become possible to identify complete genomes directly from human genetic material, without the need for prior cultivation. Nowadays, it is possible to identify both bacterial and fungal taxa in human samples by amplifying and sequencing specific genes. For bacteria, the 16S ribosomal RNA gene is commonly used, while fungi are analyzed by sequencing the 18S ribosomal RNA and the internal transcribed spacer (ITS) region [4].

This progress has significantly expanded our understanding of the composition of oral microbiomes, as well as their essential role in health and diseases status. Most studies related to oral microbiome have focused on bacteria (bacteriome); however, many other microorganisms may be present in the oral cavity, including a variety of fungal species (mycobiome). The oral mycobiome corresponds to less than 0.06% of the microorganisms in saliva and only 0.0001% in dental plaque [5].

Although fungi represent a smaller part of the microbiome compared to bacteria, they can play an important role in homeostasis and dysbiosis, contributing to the health or damage of oral mucosal and dental tissues [6]. In addition, the oral mycobiome may exert effects beyond the oral cavity, influencing the systemic health. Advances in next-generation sequencing technologies have demonstrated that oral dysbiosis and changes in community diversity can contribute to systemic inflammation, immune modulation, and endothelial dysfunction [7,8]. Fungal communities, especially *Candida* spp., have been linked to conditions such as cardiovascular diseases, adverse pregnancy outcomes, respiratory diseases [7], HIV infection progression [9], and schizophrenia [10].

Therefore, it is essential to recognize the presence and influence of the fungal component in the maintenance of oral health, which can help us identify possible relationships between fungal dysbiosis and oral and systemic diseases [6]. Certain fungal species are naturally part of the human microbiome, but many fungi can be acquired from the environment and just pass through the oral cavity. Some even fail to develop in the oral cavity because of unfavorable conditions, such as temperature or competition for nutrients with bacteria. It is therefore essential to know which fungi can multiply in the mouth, to avoid misinterpretation of mycobiome data. However, the low abundance and limited sensitivity of some detection techniques raise doubts as to whether these fungi are only passingly present or whether they are in fact commensals or possible disease causes [11]. Consequently, there is still a paucity of data on the mycobiome, especially when compared to the knowledge available on bacteria [12].

This narrative review aims to analyze the available scientific evidence on the composition, dynamics and functionality of the oral mycobiome, providing an understanding about the main factors that modulate the fungal balance in the oral cavity and the mechanisms by which dysbiosis contributes to the development of local pathological conditions. In addition, this review briefly addresses the associations between the oral mycobiome and other clinical conditions.

## 2. Strategy for Conducting the Narrative Review

In this narrative review, the literature search was conducted using the PubMed and ScienceDirect databases without date restrictions. The search strategy employed Boolean operators to combine the following descriptors: “oral mycobiome”, “oral fungi”, “oral candidiasis”, “dental caries”, “periodontal diseases”, “oral dysplasia” and “oral cancer”. The inclusion criteria comprised original studies and review articles published in English that directly addressed the composition, functionality, or clinical implications of the oral mycobiome characterized by molecular identification methods. Exclusion criteria included duplicate records, conference abstracts, editorials, studies unrelated to the scope of the review, and articles lacking sufficient scientific relevance or methodological rigor.

## 3. Healthy Oral Mycobiome

There are several studies dedicated to characterizing commensal mycobiome in healthy individuals. In these studies, the terms “central mycobiome” or “basal mycobiome” is commonly used to refer to the components of the mycobiome that remain relatively stable over time in these individuals [4,13,14].

To understand the role of the mycobiome in oral diseases, it is essential to first know its composition under healthy conditions. One of the first studies to analyze the oral mycobiome employed oral buccal samples from healthy individuals of different ethnicities with a Western diet. The oral mycobiome was characterized using a multitag pyrosequencing approach with primers targeting to the fungal internal transcribed spacer (ITS) region. In total, 74 culturable and 11 non-culturable fungal genera from the oral cavity of the studied population were found. This study also revealed between nine and 23 cultivable species per individual. From this, a set of genera associated with the basal oral mycobiome was defined, including *Alternaria*, *Aspergillus*, *Aureobasidium*, *Candida*, *Cladosporium*, *Cryptococcus*, *Dothioraceae*, *Eurotium*, *Fusarium*, *Glomus*, *Saccharomyces*, and *Teratosphaeria* [15].

Using genome sequencing techniques, later studies also identified *Malassezia*, *Irpex*, *Cytospora*/*Valsa*, *Lenzites*/*Trametes* and *Sporobolomyces*/*Sporidiobolus* as possible members of the healthy mycobiome [13,16]. Based in these studies, it is known that the basal oral mycobiome is predominantly composed of *Candida* (40–70%), followed by *Cladosporium* (2–20%), *Aureobasidium* (4–6%) and fungi from the Saccharomycetales order (13%). *Aspergillus* (2%), *Fusarium* (1–3%) and *Cryptococcus* (0.5–2%) occur in less abundance [15]. The fungal genera found in the oral cavity tend to dominate individual samples in a mutually exclusive manner, forming distinct fungal communities known as mycotypes [14,17].

Recent evidence obtained through ITS-based sequencing indicates that these mycotypes are associated with specific clinical characteristics as well as corresponding bacterial profiles. The least diverse mycotype, dominated by *Candida*, was related to an equally less diverse bacteriome, with a predominance of acidogenic and aciduric bacteria, such as *Lactobacillus* and *Propionibacterium*. On the other hand, more diverse communities dominated by *Malassezia* were associated with a more complex bacteriome, enriched with inflammatory species from the genera *Fusobacterium*, *Porphyromonas*, *Prevotella* and *Leptotrichia*, among others. In addition, the mycotypes are correlated with specific clinical characteristics. For example, *Candida*-dominated communities were more frequent in smokers, dental prosthesis users, and individuals that take corticosteroids, possess a higher plaque index and/or have active caries [14]. Therefore, in this review, we discuss specific factors that modify the oral mycobiome and lead to dysbiosis, which consequently can result in local diseases, such as oral candidiasis, dental caries, periodontal diseases and dysplasia.

## 4. Factors That Modify Oral Mycobiome

In the oral health condition, the microorganisms that compose the microbiome maintain a beneficial and balanced relationship, acting together to prevent the colonization and establishment of pathogens in the oral cavity [18]. In general, the fungi present in the microbiome remain in a commensal state, being harmless or even performing defense functions. When this microbial community is suppressed or eliminated, fungal organisms can become prominent, favoring the proliferation of pathogenic microorganisms and triggering inappropriate inflammatory or immune responses against commensal microorganisms [19].

In this context, various intrinsic and extrinsic host factors can modulate the composition and balance of the oral mycobiome, favoring both the maintenance of homeostasis and the establishment of a dysbiosis state. Intrinsic factors are mainly related to the individual’s immune status. Age is also a relevant factor: in the elderly, there is a tendency towards a reduction in the diversity of the mycobiome and greater susceptibility to oral candidiasis, possibly due to changes in salivary production and the innate immune response. In addition, genetic and hormonal characteristics can modulate the composition of the mycobiome, as evidenced by fungal alterations associated with hormonal variations during pregnancy. Among extrinsic factors, the use of medication, especially antibiotics and antifungals, can cause significant imbalances in the oral mycobiome. Prolonged use of antibiotics reduces bacterial competition, favoring the growth of fungi, mainly of the *Candida* genus. In addition, smoking and alcohol consumption are among the main behavioral factors that negatively affect the oral mycobiome [16].

In relation to intrinsic factors, patients with genetic immunological disorders generally present alterations in the oral mycobiome. Patients with defects in the Th17/IL-17 axis, present recurrent oral fungal infections. Analysis of fungal communities in the oral mucosa of these patients revealed severe dysbiosis with *Candida albicans* dominance. Active candidiasis was associated with decreased microbial diversity and enrichment of *Streptococcus oralis* and *Streptococcus mutans*, suggesting a cross-kingdom interaction of *C. albicans* with oral *streptococci* [18].

Human immunodeficiency virus (HIV) infection has also a substantial impact on the composition and balance of the oral microbiome, primarily due to immunosuppression caused by the progressive reduction in CD4^+^ T cells [20]. Studies showed that people living with HIV (PLHIV) have a significant increase in colonization by opportunistic fungi, especially species of the *Candida* genus, particularly *Candida albicans*. Other species such as *Nakaseomyces glabratus* (formerly known as *Candida glabrata*), *Candida tropicalis*, *Pichia kudriavzevii* (formerly known as *Candida krusei*), and *Candida dubliniensis* are also frequently detected in this group, especially in patients with CD4 counts below 200 cells/mm^3^ [20,21]. In addition to the increased fungal load, there is a reduction in the diversity of the oral mycobiome, characterized by the predominance of a few taxa and a decrease in the presence of commensal fungi that, under normal conditions, contribute to the oral ecological balance [20].

Another relevant aspect in PLHIV is the intensification of interactions between fungi and pathogenic bacteria. *C. albicans* can interact with *S. mutans*, *Porphyromonas gingivalis*, and other oral microorganisms, forming complex and resistant biofilms that hinder the action of the immune system and antimicrobial agents. These microbial interactions contribute to the persistence of infection and the worsening of oral manifestations [22].

Clinically, these changes in the oral mycobiome translate into a higher incidence of fungal infections, particularly oral candidiasis in its various clinical forms, which remain common manifestations of HIV infection, even after the advent of antiretroviral therapy (ART) [22]. ART, in turn, can contribute to partial recovery of the immune system and a reduction in oral fungal load. However, complete normalization of the oral mycobiome is not always achieved, and the effect of ART on fungal composition can vary depending on the therapeutic regimen, treatment adherence, and the patient’s immune status. Therefore, HIV is directly associated with oral mycobiome dysbiosis, characterized by reduced fungal diversity, increased colonization by opportunistic species, and a favored pathogenic interaction. These changes not only reflect systemic immunosuppression but can also contribute to local complications, chronic inflammation, and an increased risk of potentially malignant lesions in the oral mucosa [23].

In the context of extrinsic factors, Sajid et al. [24] conducted a pilot study to evaluate the impact of smokeless tobacco consumption (tobacco products without combustion commonly consumed in India) on the oral mycobiome by sequencing the fungal ITS1 region from oral swab samples. When comparing non-users of smokeless tobacco with users who did or did not present oral candidiasis lesions, the authors demonstrated that chronic use of smokeless tobacco is associated with significant dysbiosis of the oral mycobiome, characterized by a marked reduction in fungal load and diversity. These alterations were particularly pronounced in individuals with oral lesions, suggesting a possible association between tobacco-related mycobiome disruption and oral mucosal pathology. In a complementary investigation, Sajid et al. [25] evaluated the composition and ecological functionality of fungal communities associated with smokeless tobacco consumption. The study showed that non-users exhibited a healthier oral mycobiome profile, characterized by greater fungal diversity and predominance of *Candida* species, which accounted for approximately 40–70% of the fungal community. In contrast, smokeless tobacco users, particularly those presenting with oral lesions, showed reduced fungal richness and diversity, together with an increased relative abundance of non-*Candida* genera, such as *Pichia* (~10–15%). Collectively, these findings support the hypothesis that sustained exposure to smokeless tobacco promotes oral mycobiome dysbiosis, which may contribute to oral carcinogenesis through disruption of fungal ecological homeostasis [24,25].

In addition, various studies showed that chronic alcohol use has significant effects on the oral mycobiome, promoting changes in its composition, diversity, and ecological balance. Ethanol, the main component of alcohol, and its metabolite acetaldehyde contribute to this imbalance by promoting changes in oral pH, mucosal damage, and impaired local immunity [23]. This environment favors the proliferation of opportunistic fungi, especially *C. albicans*, *N. glabrata*, and *C. tropicalis*. The increased presence of these fungi is related to increased adhesion to the oral mucosa, a greater capacity to form biofilms, and increased expression of virulent genes [26]. The fungal growth also leads to the formation of complex oral biofilms, often involving synergistic interactions between *Candida* and pathogenic bacteria, such as *S. mutans*. Such biofilms are more resistant to the host immune response and antimicrobial treatments [27].

Thus, scientific evidence indicates that the oral mycobiome is strongly influenced by a combination of intrinsic and extrinsic factors. Understanding of these influences is fundamental not only for the prevention of oral fungal infections, but also for the development of therapeutic strategies that aim to restore and maintain oral fungal balance in different clinical and population contexts. In this scenario, many researchers have sought to identify the oral mycobiome profile under specific conditions of oral cavity, particularly in the presence of candidiasis lesions, dental caries, periodontal diseases and dysplasia or cancer (Table 1).

## 5. The Relationship Between the Mycobiome and Oral Diseases

### 5.1. Mycobiome and Oral Candidiasis

Oral candidiasis is the most common fungal infection, frequently occurring in individuals weakened by other diseases or clinical conditions, and is therefore commonly classified as an opportunistic disease [38,39]. It is predominantly caused by *C. albicans*; however, the widespread use of antifungals in recent years, both empirically and prophylactically, has contributed to a change in this scenario, favoring the emergence of other yeast species, including non-*C. albicans* species such as *N. glabratus*, *C. tropicalis*, *P. kudriavzevii*, and *C. dubliniensis*. Due to the change in the infection profile of candidiasis, it becomes necessary to improve rapid techniques for the accurate identification of species and study of the mycobiome, thus enabling correct treatment and the choice of appropriate antifungal therapy in the face of fungal infections [28,40].

Ieda et al. [29] investigated the mycobiome of patients with oral candidiasis and evaluated changes following antifungal therapy using PCR analysis combined with ITS length heterogeneity profiling. The study included 52 patients with pseudomembranous oral candidiasis and 30 healthy individuals as controls. Affected patients exhibited greater fungal species diversity and a higher fungal burden compared to controls. *C. albicans* was the most frequently identified species in both groups; however, *C. dubliniensis* was detected at a significantly higher proportion in patients with pseudomembranous oral candidiasis. Considering only LH-PCR peaks with an area ≥1% of the total, the study reported fungi such as *C. albicans*, *Rhodotorula slooffiae*, *Debaryomyces hansenii*, *Geotrichum candidum*, *Toxicocladosporium irritans*, *Ceriporia lacerata*, *Malassezia restricta*, *C. dubliniensis*, and *Rhodotorula minuta*, among others. After antifungal treatment, a marked reduction in the detection of *C. dubliniensis* was observed, and the mycological profile of patients with candidiasis became similar to that of the control group. There was a significant decrease in the total and mean number of detection signals per individual. Additionally, the detection rate of *C. albicans* increased, whereas *C. dubliniensis* became virtually undetectable [29].

Imabayashi et al. [28] investigated the oral fungal biodiversity of patients with oral candidiasis and healthy individuals, in which fungal populations were quantified using real-time PCR targeting the ITS region, and species diversity was further assessed through NGS analysis of the ITS1 region in both groups. A total of 107 fungal species were identified, with the total and mean number of species per individual being higher in the control group than in patients with oral candidiasis. *C. albicans* accounted for more than 80% of the total fungal population in both groups, and the proportion of *C. dubliniensis* was higher in patients with oral candidiasis compared to healthy individuals. In relation to other fungal species, *C. parapsilosis*, *Wallemia sebi*, *Rhodosporidium babjevae*, *P. kudriavzevii*, *Antrodiella micra*, *Cladosporium sphaerospermum*, and species belonging to the order *Sporidiobolales* were detected at significantly higher rates in patients with oral candidiasis. Conversely, *Exophiala equina*, *Cladosporium halotolerans*, and species of *Agaricomycetes* were more frequently detected in controls. Notably, some species identified in this study, including *Exophiala equina* and *Trichosporon cutaneum*, had not previously been reported as part of the oral mycobiome, likely due to limitations of earlier detection techniques. Given that *Trichosporon cutaneum* has been associated with systemic mycoses, these findings suggest that oral candidiasis may potentially contribute to the pathogenesis of systemic fungal infections [28].

Lin et al. [9] analyzed the salivary mycobiome of individuals living with HIV/AIDS at different stages of infection, including some patients with oral candidiasis, and compared these profiles with those of HIV-negative controls. Using next-generation sequencing (ITS region), the authors observed that mycobiome diversity in HIV-positive individuals decreased progressively as the disease advanced. The lowest level of diversity was identified in patients at HIV stage 3, corresponding to the most advanced phase of infection (AIDS). Additionally, it was observed a transient increase during the early stage of infection (HIV stage 0) compared with HIV-negative controls, followed by a gradual decline as the infection progressed. These findings suggest that salivary mycobiome analysis may serve as a potential non-invasive biomarker for monitoring HIV progression and may also support the development of future diagnostic and preventive strategies targeting oral fungal infections [9].

Lin et al. [9] also emphasized the dynamic changes in the salivary mycobiome throughout the progression of HIV infection. Regarding taxonomic composition, it was observed that at the phylum level, all groups were predominantly composed of Ascomycota, followed by Basidiomycota. At the genus level, *Alternaria* and *Talaromyces* showed low abundance in the control group but increased following HIV infection, suggesting that these genera may be present at different stages of the disease. In contrast, *Coniosporium* and *Sarcinomyces* exhibited reduced abundance after infection. Additionally, *Debaryomyces* and *Talaromyces* demonstrated a moderate positive correlation with CD4^+^ T-cell counts, whereas *Penicillium* showed a negative correlation with viral load. There were no significant differences in mycobiome composition between individuals living with HIV/AIDS with and without candidiasis. These findings indicate that certain oral fungal populations in HIV-infected individuals may be influenced by both CD4^+^ T-cell counts and viral load levels [9].

These findings suggest that mycobiome characterization enables understanding of fungal population dynamic in health and disease, indicating that a marked increase in a particular species, accompanied by a relative decrease in others, may promote the development of oral candidiasis [41,42].

### 5.2. Mycobiome and Dental Caries

Dental caries is a multifactorial disease characterized by the demineralization of hard dental tissues resulting from acids produced by the fermentation of dietary carbohydrates by oral microorganisms present in the dental biofilm [43,44]. The role of dental biofilm bacteria in the etiology of caries is well established, and over the past two decades, the application of molecular techniques has demonstrated the high diversity of oral bacterium communities, as well as their association with disease development [31,45].

Since advances in molecular techniques have significantly expanded the list of microorganisms potentially involved in the pathogenesis of caries, the role of the oral mycobiome in dental caries has attracted increasing scientific interest. Several studies have investigated the diversity of the fungal community in relation to the presence or absence of caries, revealing significant differences in mycobiome composition according to the host’s oral health status [6,45].

O’Connell et al. [31] aimed to clarify the complex microbial interactions involved in the etiology and progression of early childhood caries. Using ITS region sequencing, they generated taxonomic profiles from supragingival plaque samples collected from children under different clinical conditions: (1) caries-free, (2) with active caries confined to enamel, and (3) with active caries involving dentin. A total of 139 fungal species was identified, with *C. albicans* being the most abundant, followed by *C. dubliniensis.* The researchers found significant differences between the supragingival plaque mycobiome of caries-free children and that of children with lesions extending into dentin. Species such as *C. albicans*, *C. dubliniensis*, *Neoscytalidium oryzae*, and unclassified species of the genus *Microdochium* were associated with caries, whereas 12 other taxa were linked to oral health. Notably, *C. dubliniensis* showed a progressive increase as caries advanced into dentin. In contrast, four fungal genera associated with oral health, *Debaryomyces*, *Rhodotorula*, *Aureobasidium*, and *Aspergillus*, demonstrated potential antagonistic activity against *Streptococcus mutans*, a key cariogenic bacterium [31].

In the study conducted by Jesus et al. [46], the oral mycobiome of 40 children with caries and 40 caries-free children was compared using next-generation sequencing (NGS) of the ITS1 region of rRNA. The results demonstrated relevant differences in the variety of species present within each group. The genus *Candida* was the most abundant, being detected in 68 of the 73 samples, with higher prevalence in the caries group. At the species level, *C. dubliniensis* was the dominant species among affected children, whereas no significant difference in the abundance of *C. albicans* was observed between children with and without caries. Additionally, species belonging to the genera *Mycosphaerella*, *Cyberlindnera*, and *Trichosporon* showed differences in abundance when comparing caries-free and caries-affected groups [46].

Fechney et al. [30] evaluated the composition of the oral mycobiome using next-generation sequencing (NGS) of the ITS2 region, amplified from dental plaque samples collected from 17 children. In total, 23 genera and 46 fungal species were detected across all plaque samples, with *C. albicans*, *Naganishia diffluens*, *Rhodotorula mucilaginosa*, and *Malassezia globosa* being the most abundant species. Although the overall diversity of fungi was similar regardless of the presence of caries, the disease was found to be associated with changes in the abundance of specific fungal species, corroborating with other studies [30,47].

Tu et al. [32] used V3–V4 16S rRNA and ITS1 rRNA gene amplicon sequencing to analyze salivary bacterial and fungal profiles in children with and without caries. In both groups, *Candida* was the most abundant fungal genus, followed by *Cladosporium*, *Aspergillus*, *Wallemia*, and *Malassezia*. A significant alteration in the salivary fungal community was observed in children with caries compared to the caries-free control group, and the fungal type influenced the structure of the bacterial community. As for health-related taxa, fungal genus *Leptospora* was positively associated with the bacterial genus *Streptococcus.* Regarding caries-related taxa, *Hannaella*, *Vishniacozyma* and *Lepista* were found to be positively associated with the bacterial genera *Actinomyces* or *Bergeyella* [32].

Du et al. [48] investigated the role of *C. albicans* in the etiology of dental caries from an ecological perspective. Through in vitro and in vivo experimental models, the study showed that the presence of *C. albicans* significantly altered the composition of the bacterial community within the biofilm, favoring acidogenic and aciduric microorganisms associated with enamel demineralization. The interaction between the fungus and cariogenic bacteria enhanced the formation of more structured and metabolically active biofilms with greater acid-producing capacity. Moreover, colonization by *C. albicans* was associated with increased severity of carious lesions. These findings indicate that *C. albicans* is not merely an opportunistic colonizer, but an ecological modulator capable of reshaping the oral microbiota toward a dysbiotic state, thereby increasing the cariogenic potential of the biofilm. The study reinforces the concept of dental caries as a polymicrobial disease and highlights the importance of considering the oral mycobiome in understanding its pathogenesis and in the development of preventive and therapeutic strategies [48].

In summary, there is clear evidence that the mycobiome plays a role in the development of carious lesions, particularly in the more advanced stages of the disease. However, further studies are needed to better understand how fungi interact with oral bacteria and how these interactions may enhance the virulence of the polymicrobial oral biofilm in dental caries [32,46].

### 5.3. Mycobiome and Periodontal Diseases

Periodontitis is a chronic inflammatory disease that affects the periodontium, the tissues responsible for surrounding and supporting the teeth. It is highly prevalent among adults and represents the leading cause of tooth loss worldwide. Its etiology is associated with a synergistic and dysbiotic microbial community present in the subgingival biofilm, in which keystone pathogens play a central role in disrupting tissue homeostasis. In addition to bacteria, fungi may also be an important component of the subgingival biofilm. The combined action of these microorganisms, organized within polymicrobial communities, influences the clinical progression of the infection and may impact therapeutic strategies. Therefore, a deeper understanding of the involvement of the mycobiome in periodontitis is essential to improve treatment approaches, especially considering that most studies have predominantly focused on the bacteriome [33,49,50,51].

The oral mycobiome of individuals with and without periodontal disease was characterized using NGS of the ITS region in the study conducted by Peters et al. [33]. Across all analyzed samples, at least four phyla, Ascomycota, Basidiomycota, Glomeromycotan and Chytridiomycota, were identified, as well as a minimum of 81 genera and 154 fungal species. *Candida* and *Aspergillus* were the most frequently detected genera, being isolated in 100% of the participants, followed by *Penicillium* (97%), *Schizophyllum* (93%), *Rhodotorula* (90%), and *Gibberella* (83%). At the species level, three taxa were observed in all individuals: uncultured Dikarya, *Candida* spp., and *Aspergillus niger*. Among these, *Aspergillus niger* and *Candida* spp. were the most abundant species in the samples. *Candida* genus showed higher relative abundance in participants with periodontal disease compared to periodontally healthy individuals, although this difference was not statistically significant. This study was the first to characterize the oral mycobiome in relation to periodontal disease using a comprehensive targeted sequencing approach [33].

The diversity of the mycobiome was assessed among individuals without periodontitis, with mild periodontitis, and with moderate to severe periodontitis in the study conducted by Acosta-Pagán et al. [35]. The most abundant genera across all categories included *Candida*, *Saccharomyces*, *Rigidoporus*, *Aspergillus*, and *Trametes*, with *Aspergillus* being among the most representative. Species-level characterization of the mycobiome was performed comparing healthy participants and those with periodontal disease. The healthy mycobiome was predominantly composed of *Rigidoporus vinctus*, *C. parapsilosis*, and *C. albicans*, while species such as *C. dubliniensis*, *Saccharomyces cerevisiae*, and *Trametes elegans* were detected at lower levels. In contrast, participants presenting any degree of periodontal disease exhibited a higher relative abundance of *C. tropicalis*, *Saccharomyces cerevisiae*, *Rigidoporus vinctus*, and *Irpex lactus* [35].

Yao Hu et al. [52] conducted a narrative review exploring the role of fungi in periodontitis, particularly species of the genus *Candida.* The review analyzed clinical articles, observational studies, and trials addressing the presence and impact of *Candida* in periodontal tissues. The review shows that several *Candida* species are frequently detected in the oral cavity of individuals with periodontitis and suggests that these yeasts may not merely be opportunistic colonizers but may also act as co-factors in the inflammatory process. The species most cited in the literature include *C. albicans*, which is the most prevalent and most extensively studied in this context. In addition, other non-*albicans Candida* species have also been reported in association with periodontitis, such as *N. glabratus*, *C. tropicalis*, *and C. parapsilosis*. These species vary in their virulence and biofilm-forming capacity, and may interact with periodontopathogenic bacteria, thereby influencing the periodontal pocket microenvironment. In addition, the review reports potential mechanisms by which *Candida* may contribute to inflammation and tissue destruction, including the formation of mixed biofilms with bacteria, the induction of dysregulated immune responses, and the production of fungal metabolites that may amplify periodontal inflammation. The authors also suggest that, given the growing evidence of fungal involvement in periodontitis, *Candida* species could represent therapeutic targets in ecological management strategies for the disease [52].

Based on the literature surveyed, the main fungal genera isolated from the oral microbiome of individuals with periodontitis appear to coincide with those present in the central mycobiome. However, the precise role of fungi in the onset and progression of periodontitis has yet to be fully elucidated [53].

### 5.4. Mycobiome and Alterations of the Oral Mucosa

In recent years, growing scientific attention has been directed toward understanding the role of the oral mycobiome in the development of oral mucosal alterations, and dysbiotic fungal communities have been consistently identified in these conditions. Among the most common alterations associated with changes in the oral mycobiome are leukoplakia, dysplasia, and carcinoma. Increasing evidence suggests that microbial dysbiosis is not merely a consequence of the tissue changes linked to these lesions, but may precede their onset and actively contribute to their development and progression [54,55,56].

A deeper understanding of microbial dysbiosis concepts, together with advances in high-throughput molecular sequencing technologies, has enabled a more detailed microbiological characterization of clinical conditions, highlighting the association between fungal presence and oral mucosal alterations [57]. In this context, several clinical studies have sought to investigate and characterize the fungal communities associated with various oral mucosal alterations.

In this context, Li et al. [58] focused on oral lichen planus (OLP), a chronic inflammatory condition of the oral mucosa that affects approximately 0.5% to 2% of the adult population. Their study was conducted in individuals with reticular OLP (*n* = 17) and erosive OLP (*n* = 18), which were compared to healthy controls (*n* = 18). The oral mycobiome was analyzed using NGS of the ITS2 region. Fungal community composition was assessed at different taxonomic levels. At the phylum level, significant differences were observed between the two predominant phyla: Ascomycota (59.03% in healthy controls, 69.58% in reticular OLP, and 68.22% in erosive OLP) and Basidiomycota (15.62%, 13.46%, and 7.33%, respectively). At the genus level, a total of 280 genera was detected. The relative abundance of *Candida* and *Aspergillus* was significantly increased in the reticular OLP group compared to healthy controls. In contrast, the genus *Trichosporon* was notably more abundant in healthy individuals than in those with erosive OLP. Overall, the oral fungal community exhibited reduced enrichment and lower complexity in OLP patients compared to healthy individuals. The authors concluded that the oral cavities of OLP patients harbor a dysbiotic and less complex mycobiome, suggesting that fungal dysbiosis is associated with disease progression and severity [58].

The oral mycobiome associated with oral squamous cell carcinoma have also been investigated through quantification of the internal transcribed spacer 2 (ITS2). Perera et al. [36] analyzed oral samples from 25 individuals with carcinoma and 27 controls. A total of 364 species belonging to 162 genera and two phyla were identified. The phyla Ascomycota and Basidiomycota accounted for 78.4% and 21.6% of the mycobiome, respectively. At the genus level, *Candida* was detected in 100% of the samples and represented 48% of the average mycobiome composition. The genera *Malassezia*, *Cladosporium*, and *Aspergillus* were identified in at least 75% of the samples, with mean relative abundances of 11%, 6.1%, and 3.7%, respectively. At the species level, *C. albicans* was present in all samples, with a mean relative abundance of 44.4%. *Malassezia restricta*, *Aspergillus penicillioides*, and *Malassezia globosa* were detected in 83%, 70.2%, and 68.1% of samples, corresponding to 3.2%, 2.2%, and 4.2% of the average mycobiome, respectively. Control samples showed significantly greater species richness and alpha diversity compared to the carcinoma group. *C. albicans*, *C. etchellsii*, and a potentially novel species related to *Hannaella luteola* were significantly enriched in the carcinoma group. Although *C. albicans* was detected in 100% of samples, its mean relative abundance in carcinoma cases was twice that observed in controls. The *H. luteola*-like species was detected in 20% of carcinoma samples and was absent in controls [36].

The prevalence of *Candida* species in the saliva of patients with oral squamous cell carcinoma, with potentially malignant oral lesions, and healthy individuals was evaluated using the PCR-RFLP method by Sankari et al. [59]. The findings revealed a predominance of non-albicans *Candida* species across all three groups. In the oral squamous cell carcinoma group, the non-albicans species identified were: *P. kudriavzevii* (21%), *C. tropicalis* (21%), *Pichia anomala* (21%), *C. famata* (17%), *N. glabratus* (5%), *C. rugosa* (6%), *C. orthopsilosis* (4%), *C. kefyr* (2%), *C. lusitaniae* (2%), and *C. stellatoides* (1%). Among patients with potentially malignant lesions, the isolated species included: *P. anomala* (formerly *C. pelliculosa*) (33%), *P. kudriavzevii* (27%), *C. tropicalis* (10%), *C. famata* (9%), *C. rugosa* (7%), *C. guilliermondii* (5%), *C. parapsilosis* (3%), *C. orthopsilosis* (3%), *C. glabrata* (1%), *C. stellatoides* (1%), and *C. intermedia* (1%). In healthy controls, the non-albicans species detected were *P. anomala* (40%), *C. tropicalis* (24%), *P. kudriavzevii* (17%), *C. guilliermondii* (4%), *C. parapsilosis* (4%), *C. orthopsilosis* (4%), *C. dubliniensis* (4%), and *C. nivariensis* (4%). A notably high prevalence of *P. anomala* was observed among healthy individuals (43.5%), followed by patients with potentially malignant lesions (33%) and those with oral squamous cell carcinoma (21%) [59].

The high frequency of oral candidiasis in patients with oral squamous cell carcinoma and potentially malignant lesions suggests a possible association between fungal infection and these clinical conditions. These findings emphasize the importance of *Candida* suppression to prevent opportunistic infections and potentially reduce the risk of malignant transformation of potentially malignant lesions into oral squamous cell carcinoma [59,60,61].

Although the presence of *Candida* is frequently observed in potentially malignant oral lesions and in established tumors, it is unclear whether this microorganism plays an active role in carcinogenesis or merely colonizes previously altered tissues. To address this question, Wang et al. [62] investigated the role of *C. albicans* in the progression of oral squamous cell carcinoma, focusing on the immunological mechanisms involved in the interaction between the fungus and the tumor microenvironment. Using in vivo experimental models, the authors demonstrated that colonization by *C. albicans* significantly enhanced tumor growth. The identified mechanism involved activation of the interleukin-17A (IL-17A) pathway and its receptor, IL-17RA. Fungal infection stimulated IL-17A production, a cytokine classically associated with antifungal immune responses, which in this context contributed to the establishment of a tumor-promoting inflammatory environment [62].

In a review article, Yu et al. [63] examined recent advances in understanding the interaction between *C. albicans* and various cancers. The authors highlight that *C. albicans* may contribute to carcinogenesis through multiple mechanisms, including the induction of chronic inflammation, production of carcinogenic metabolites such as acetaldehyde, activation of pro-tumor signaling pathways (such as NF-κB and STAT3), and modulation of the tumor microenvironment. Moreover, the filamentous (hyphal) form of the fungus is considered particularly significant due to its invasive capacity and heightened inflammatory potential [63].

Taken together, these findings highlight the complex and multifaceted contribution of fungal communities to oral mucosal alterations. Although *Candida* remains the most extensively studied genus, growing evidence supports a broader model in which multiple fungal taxa, together with the bacterial microbiota and host-related factors, interact to drive the initiation and progression of these alterations.

## 6. Associations Between the Oral Mycobiome and Other Clinical Conditions

Besides the oral diseases discussed above, dysbiosis of the oral mycobiome has also been associated with other oral pathological conditions and even systemic diseases, particularly those commonly observed in pediatric patients, such as orthodontic therapy, cleft lip/palate, autism spectrum disorder (ASD), and asthma [8]. Pérez-Losada et al. [64] investigated the oral mycobiome of 349 participants (313 children and adolescents and 36 adults), distributed in the following groups: allergic rhinitis, allergic rhinitis associated with asthma, asthma, and healthy controls. The samples were collected by swab of the jugal mucosa and submitted to sequencing of the ITS1 and ITS2 regions on the Illumina MiSeq platform. The results showed that Ascomycota and Basidiomycota were the predominant phyla but showed significant differences between individuals with allergic rhinitis and controls. Among the most abundant genera, *Cladosporium*, *Aspergillus*, *Aleurina*, *Candida*, and *Rhodotorula* exhibited differential abundance in the groups with allergic disease, while no significant differences were observed between the different groups of respiratory diseases. In addition, patients with respiratory diseases had higher alpha diversity and significant changes in beta diversity compared to healthy controls, indicating changes in the composition and structure of the oral mycobiome. The authors concluded that allergic rhinitis, alone or associated with asthma, is related to changes in the taxonomic composition, diversity, and functional organization of the oral mycobiome, suggesting its possible involvement in the pathophysiology of allergic respiratory diseases [64].

In adults, recent studies have demonstrated a significant association between oral mycobiome dysbiosis and specific clinical conditions, including endotracheal intubation, SARS-CoV-2 infection, and chronic environmental exposure to heavy metals.

Song et al. [65] simultaneously evaluated the bacteriome and the oral mycobiome of four adult patients intubated in an intensive care unit, using samples obtained by swab of the jugal mucosa collected in the period from 0th to 12th day of hospitalization. The DNA was subjected to sequencing of the V1–V2 regions of the 16S rRNA and ITS2 genes. The analyses based on ITS2 showed that intubation was associated with significant changes in the composition of the oral mycobiome, with a reduction in fungal diversity and a predominance of opportunistic fungi. The genus *Candida* showed a significant increase in relation to healthy individuals, with *C. albicans* being the dominant species in the samples of intubated patients, while *N. glabratus*, *C. parapsilosis*, *Candida sake* and *C. tropicalis* were detected in lower abundance. In addition, changes in the relative abundance of other genera were observed, including *Malassezia*, *Cladosporium*, *Saccharomyces*, and *Aspergillus*, evidencing an imbalance of the oral fungal community during ICU admission. The co-occurrence analyses also revealed modifications in the interactions between fungi and bacteria, suggesting that the enrichment of *C. albicans* and the reduction in mycobiome diversity may favor colonization by opportunistic microorganisms and contribute to the development of ventilator-associated respiratory infections [65].

Hu et al. [66] evaluated the oral mycobiome of patients with SARS-CoV-2 by sequencing the ITS2 region, using lingual lining samples from confirmed, suspected, recovered patients, and healthy controls. Regarding the taxonomic composition, the phyla Ascomycota, Basidiomycota and Mucoromycota corresponded to approximately 95% of the sequences, being the most abundant in all groups. However, significant dysbiosis was observed in the SARS-CoV-2 group, with a relative increase in Ascomycota and Zoopagomycota and a reduction in Basidiomycota, Mucoromycota and Rozellomycota. At the genus level, an enrichment of *Acrodictys*, *Candida*, *Saccharomyces*, and *Simplicillium* was observed in patients with COVID-19 disease and a reduction in *Malassezia*, *Cladosporium* and *Cryptococcus*. At the species level, several alterations were identified, including an increase in *C. albicans* in the infected group and a decrease in other fungi such as *Zanclospora jonesii*, *Cryptococcus longus* and *Diversispora spurca*. Alpha and beta diversity analyses demonstrated significant differences between COVID-19 patients and controls, with clear separation between the groups, indicating oral mycobiome dysbiosis associated with SARS-CoV-2 infection [66].

Li et al. [67] investigated the effects of chronic environmental exposure to heavy metals on the oral mycobiome through sequencing of the ITS1 region. The study included 136 individuals aged between 42 and 72 years, living in two regions of Gansu province, China, with a group from Baiyin, an area characterized by high environmental contamination due to mining activities, and a control group from Lanzhou, a region with low contamination. The exposure was confirmed by inductively coupled plasma mass spectrometry (ICP-MS), which showed significantly higher concentrations of cadmium, copper, zinc, lead, mercury, molybdenum and antimony in the exposed region. The results showed marked changes in the composition of the fungal community, with a predominance of the genus *Apiotrichum* among the exposed individuals, followed by *Cutaneotrichosporon*, while the genera *Candida* and *Aspergillus* were the most abundant in the control group and showed a significant reduction in the population exposed to heavy metals. In addition, the genera *Debaryomyces*, *Wallemia*, and *Saccharomyces* also showed significant differences between the groups. The species-level analysis showed a significant increase in *Apiotrichum montevideoense*, *Cutaneotrichosporon curvatus* and *Cutaneotrichosporon cutaneum* in individuals exposed to the metals. Considering that species of the genus *Cutaneotrichosporon* are recognized as opportunistic fungi associated with human hosts, these findings suggest that environmental exposure favors the enrichment of taxa with pathogenic potential [67].

## 7. Limitations and Future Perspectives

Although this review focused on oral diseases, there is substantial evidence that the oral mycobiome may influence a variety of systemic diseases, which were only briefly explored in this review and should be addressed more comprehensively in future studies. Future research should also include larger sample sizes, longer follow-up periods, and the analysis of a broader range of variables. Furthermore, it is essential to invest in the development and continuous updating of fungal ribosomal databases to enable a more comprehensive characterization of the oral mycobiome. Such advances may provide new insights into the processes of oral health and disease, contributing to the identification of innovative preventive and therapeutic strategies.

## 8. Conclusions

Overall, the basal oral mycobiome of healthy individuals is composed of members of the genus *Candida*, followed by *Cladosporium*, *Aureobasidium* and *Saccharomyces*. The genera *Aspergillus*, *Fusarium* and *Cryptococcus* are also detected in oral health conditions, although they generally present at lower abundances.

**Figure 1:**
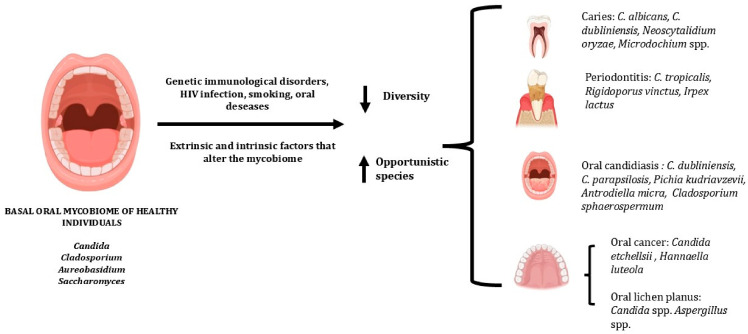
Representative patterns of fungal community composition observed under healthy versus diseased conditions. Created by the authors using BioRender.com.

Various intrinsic and extrinsic host factors can modulate the composition of the oral mycobiome, favoring a state of dysbiosis. Factors such as genetic immunological disorders, HIV infection and smoking can reduce the diversity of the oral mycobiome, promoting colonization by opportunistic species and contributing to the developing of oral diseases. *C. albicans* remains the most prevalent species in both healthy and diseased states; however, individuals with oral candidiasis show a higher detection of *C. dubliniensis*, *C. parapsilosis*, *Pichia kudriavzevii*, *Antrodiella micra* and *Cladosporium sphaerospermum*. Recently, fungal species not previously reported as part of the oral mycobiome, such as *Exophiala equina* and *Trichosporon cutaneum*, have been detected in the oral cavity of patients with oral candidiasis. Given that *Trichosporon cutaneum* has been associated with systemic mycoses, these findings raise the possibility that oral candidiasis may contribute to the pathogenesis of systemic fungal infections. In addition to *C. albicans*, metagenomic techniques have enabled the identification of several other fungi in dental caries (*C. dubliniensis*, *Neoscytalidium oryzae* and species of the genus *Microdochium*), periodontal diseases (*C. tropicalis*, *Rigidoporus vinctus*, and *Irpex lactus*) and oral cancer (*Candida etchellsii* and a potentially novel species related to *Hannaella luteola*) (Figure 1).

Although this review focused on oral diseases, there is growing evidence that the dysbiosis of the oral mycobiome is associated with a wide range of other clinical conditions, including autism spectrum disorder (ASD), asthma, endotracheal intubation, SARS-CoV-2 infection, and chronic environmental exposure to heavy metals.

This study highlights the relevance of the oral mycobiome as an essential component of human health, demonstrating that fungal communities are not merely passive colonizers but may be involved in the development of several oral diseases. Therefore, recognizing mycobiome dynamics contributes to a more integrated understanding of oral diseases, moving beyond an approach focused exclusively on the bacteriome.

## Figures and Tables

**Table 1:** Fungal species identified in the oral cavity according to clinical conditions and molecular identification methods.

Clinical Condition	Main Microorganisms Identified	Clinical Sample	Molecular Analysis	Country	Reference
Oral Candidiasis	*Candida albicans* *Candida dubliniensis* *Candida tropicalis* *Trichosporon cutaneum* *Exophiala equina*	Oral swab of injured areas	NGS of the ITS region	Japan	[28]
Oral Candidiasis	*Candida albicans* *Candida dubliniensis* *Malassezia restricta* *Toxicocladosporium irritans*	Oral swab of injured areas	LH-PCR of the ITS regions	Japan	[29]
Dental caries	*Candida albicans* *Candida sake* *Cryptococcus neoformans*	Samples of supragingival plaque	NGS of the ITS-2 region	USA	[6]
Dental caries	*Candida albicans* *Alternaria eichhorniae* *Cladosporium cladosporioides* *Malassezia globosa*	Samples of supragingival plaque	NGS of the ITS-2 region	Australia	[30]
Dental caries	*Candida albicans* *Candida dubliniensis* *Neoscytalidium oryzae* *Microdochium* spp.	Samples of supragingival plaque	NGS of the ITS-1 region	USA	[31]
Dental caries	*Candida* spp. *Malassezia* spp. *Aspergillus* spp. *Cladosporium* spp.	Saliva samples	NGS of the ITS-1 region	China	[32]
Periodontal diseases	*Candida* spp. *Aspergillus* spp. *Penicillium* spp. *Schizophyllum* spp. *Rhodotorula* spp. *Gibberella* spp.	Saliva samples	NGS of the ITS region	USA	[33]
Periodontal diseases	*Candida albicans* *Candida dublinensis* *Candida intermedia* *Candida parapsilosis* *Candida tropicalis* *Candida zeylanoides* *Cryptococcus* spp. *Aspergillus* spp.	Saliva samples	NGS of the ITS-1 region	USA	[34]
Periodontal Diseases	*Candida albicans* *Saccharomyces cerevisiae* *Rigidoporus vinctus* *Aspergillus penicilloides*	Saliva samples	NGS of the ITS-2 region	Puerto Rico	[35]
Squamous-cell carcinoma	*Candida albicans* *Candida etchellsii* *Hannaella luteola* *Hanseniaspora uvarum* *Malassezia restricta* *Aspergillus tamarii*	Tissue biopsies	NGS of the ITS-2 region	Sri Lanka	[36]
Squamous-cell carcinoma	*Candida tropicalis* *Acremonium exuviarum* *Aspergillus fumigatus* *Aspergillus sclerotiorum* *Penicillium cryptum* *Aspergillus ochraceopetaliformis*	Buccal swab samples	NGS of the ITS-1 region	China	[37]

Caption: Length Heterogeneity Polymerase Chain Reaction (LH-PCR), next-generation sequencing (NGS).

## Data Availability

No new data were created or analyzed in this study. The original contributions presented in this study are included in the PubMed database. For further information, please contact the corresponding authors.
